# Gasping in Response to Basic Resuscitation Efforts: Observation in a Swine Model of Cardiac Arrest

**DOI:** 10.1155/2010/351638

**Published:** 2010-05-31

**Authors:** Mathias Zuercher, Gordon A. Ewy, Charles W. Otto, Ronald W. Hilwig, Bentley J. Bobrow, Lani Clark, Vatsal Chikani, Arthur B. Sanders, Robert A. Berg, Karl B. Kern

**Affiliations:** ^1^Department of Anaesthesia, University Hospital Basel, CH 4031, Basel, Switzerland; ^2^Sarver Heart Center, University of Arizona, Tucson, AZ 85724, USA; ^3^Department of Medicine, College of Medicine, University of Arizona, Tucson, AZ 85724, USA; ^4^Department of Anesthesiology, College of Medicine, University of Arizona, Tucson, AZ 85724, USA; ^5^Arizona Department of Health Services Bureau of Emergency Medical Services and Trauma Systems, Phoenix, AZ 85007, USA; ^6^Department of Emergency Medicine, Maricopa County Medical Center, Phoenix, AZ 85007, USA; ^7^Department of Emergency Medicine, College of Medicine, University of Arizona, Tucson, AZ 85724, USA; ^8^Department of Pediatrics, College of Medicine, University of Arizona, Tucson, AZ 85724, USA

## Abstract

*Objective*. To analyze the effect of basic resuscitation efforts on gasping and of gasping on survival. *Methods*. This is secondary analysis of a previously reported study comparing continuous chest compressions (CCC CPR) versus chest compressions plus ventilation (30:2 CPR) on survival. 64 swine were randomized to 1 of these 2 basic CPR approaches after either short (3 or 4 minutes) or long (5 or 6 minutes) durations of untreated VF. At 12 minutes of VF, all received the same Guidelines 2005 Advanced Cardiac Life Support. Neurologically status was evaluated at 24 hours. A score of 1 is normal, 2 is abnormal, such as not eating or drinking normally, unsteady gait, or slight resistance to restraint, 3 severely abnormal, where the animal is recumbent and unable to stand, 4 is comatose, and 5 is dead. For this analysis a neurological outcome score of 1 or 2 was classified as “good”, and a score of 3, 4, or 5 was classified as “poor.” *Results*. Gasping was more likely to continue or if absent, to resume in the animals with short durations of untreated VF before basic resuscitation efforts. With long durations of untreated VF, the frequency of gasping and survival was better in swine receiving CCC CPR. In the absence of frequent gasping, intact survival was rare in the long duration of untreated VF group. *Conclusions*. Gasping is an important phenomenon during basic resuscitation efforts for VF arrest and in this model was more frequent with CCC-CPR.

## 1. Introduction

Gasping, also referred to as agonal breathing, often follows cardiac arrest [1–10]. It is a well investigated phenomenon, having been observed in all mammals. Gasping is an abnormal ventilatory activity considered to be an “auto-resuscitative” phenomenon [11]. Gasping probably occurs as a response of poor perfusion and/or hypoxia of the brain.

Investigators at Weil's Institute of Critical Care Medicine and others have shown several hemodynamic benefits of gasping. Gasping inspirations decrease intrathoracic and right atrial pressures resulting in a pressure gradient that promotes venous return to the heart [1]. Gasping expirations increase intrathoracic, aortic, pressures and coronary perfusion pressure, thereby improving blood flow [4, 11]. Others have confirmed these findings and have shown a decrease in intracranial pressure with gasping and a concomitant increase in cerebral perfusion pressure [12]. Gasping has been shown to be positively associated with improved outcome in animal models [3, 4, 11, 13]. 

Gasping is also an important phenomenon in humans suffering from cardiac arrest, with a reported occurrence of 55% in patients with witnessed out-of-hospital cardiac arrest [2]. In a study of patients with out-of-hospital cardiac arrest, 39% of individuals who gasped while receiving bystander resuscitation efforts survived to hospital discharge compared with only 9% of individuals who did not gasp while receiving bystander resuscitation efforts [14]. 

The aim of the present study was to analyze the influence of the type of basic resuscitation efforts on gasping and survival following ventricular fibrillation (VF) in a swine model of out-of-hospital cardiac arrest.

## 2. Methods

This study was conducted with the approval of the University of Arizona Institutional Animal Care and Use Committee in accordance with the guidelines set forth in the “Position of the American Heart Association on Research Animal Use.” This paper is an analysis of gasping in animals previously reported comparing two basic resuscitation protocols with regard to 24-hour neurological outcome [15]. The protocol was designed to simulate an out-of-hospital cardiac arrest responded by a single rescuer prior to the arrival of Emergency Medical Service (EMS), who then provided advanced cardiac resuscitation starting at 12 minutes. The duration of untreated VF was varied, again to simulate variable reaction times from cardiac arrest to the initiation of resuscitation efforts.

The animal preparation and experimental protocol has been published previously [15]. In summary, 64 swine (28 ± 4 kg) were anesthetized by inhalation of 5% isoflurane in oxygen. An endotracheal tube was placed, and anesthesia was maintained using 1.5%–3% isoflurane in ambient air. Ventilation was provided by a rate- and volume-regulated ventilator (Narkomed 2A, North American Drager, Telford, PA) and was adjusted to maintain an end-tidal CO_2_ pressure (EtCO_2_) of 40 ± 3 mmHg. Vascular introducer sheaths (5–7F, Cordis Corp., Miami, FL) were placed by sterile cutdown procedures, and solid state pressure transducers (MPC-500, Millar Instruments, Houston, TX) were positioned into the descending aorta and the right atrium. Electrocardiographic leads were placed. An infrared capnometer (47210A, Hewlett Packard Co., Palo Alto, CA) and a pneumotachometer (MP45–871, Validyne Engineering Corp., Northridge, CA) were used to measure EtCO_2_ and air flow. Defibrillator pads (Quik-Combo, Medtronic, PhysioControl, Redmond, WA) were adhered to the chest. All mentioned parameters were continuously displayed on a recording system (Gould Ponemah Physiology Platform, Model P3 Plus, LDS Life Science, Valley View, OH) and stored on a laptop computer for analysis.

After collection of baseline data, VF was induced by alternate current via temporarily placed pacing electrode. Continuous positive pressure ventilations were discontinued after induction of VF. The animals were assigned to one of four different untreated VF times (3, 4, 5 or 6 minutes) and were subsequently randomized to receive basic resuscitation using the continuous chest compressions (CCC) or the 30 chest compressions followed by 30 : 2 basic life support protocols. The 30 : 2 protocol interrupted each 30 chest compressions for a realistic 16 seconds to simulate the time required for a single bystander to deliver two mouth-to-mouth ventilations [16]. Basic resuscitation efforts continued until simulated emergency medical service (EMS) arrival, 12 minutes after VF induction. A single biphasic defibrillation shock (150 J) was delivered at this time, and the 2005 Guidelines Advanced Cardiac Life Support was begun in both groups. Ventilations with 100% oxygen were delivered manually by Ambu bag. Animals that had a positive return of spontaneous circulation (ROSC) were reconnected to the ventilator and underwent a one-hour intensive care period. The animals were allowed to recover from anesthesia and were placed in observation cages. At 24 hours postresuscitation, a neurological examination was performed as previously described [17–19]. Briefly, a score of 1 is normal, 2 is abnormal, such as not eating or drinking normally, unsteady gait, or slight resistance to restraint, 3 severely abnormal, where the animal is recumbent and unable to stand and only partially responsive to stimuli, 4 is comatose, and 5 is dead. For the analysis herein reported, neurological outcome with a score of 1 or 2 was classified as “good” and a score of 3, 4, or 5 was classified as “poor.” After the neurological examination the animals were euthanized.

**Figure 1:**
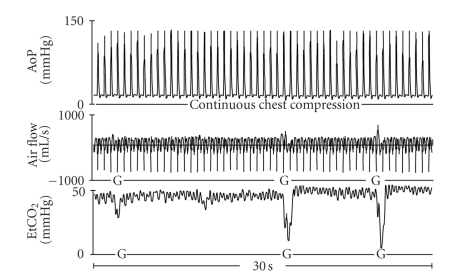
Graphic recordings of aortic pressure (AoP), air flow, and end-tidal CO_2_ (EtCO_2_) of a swine in the continuous chest compression (CCC) group. G marks a gasp.

**Figure 2:**
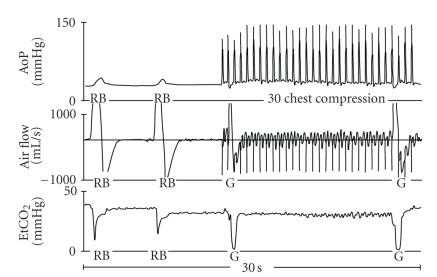
Graphic recordings of aortic pressure (AoP), air flow and end-tidal CO_2_ (EtCO_2_) of a swine in the 30 : 2-CPR group. RB indicates rescue breathing produced by exhaled air. G marks a gasp.

For this analysis, the animals with 3- or 4-minute intervals of untreated VF were combined (short VF group) and compared with the animals with 5- or 6-minute durations of untreated VF (long VF group). Gasping has been defined as an abrupt transient inspiratory effort [20]. The present analysis considered any abrupt inspiratory effort followed by an abrupt expiratory air flow measured by the pneumotachograph, which was associated with a corresponding decrease of at least 25% of the EtCO_2,_ as a gasp. Since pneumotachographs and main stream infrared capnometers are very sensitive measurement devices, this definition was used to avoid over interpretation of irrelevant signals or artefacts. Figures 1 and 2 are simultaneous recordings of aortic pressures (mmHg), air flow (mL/sec), and end-tidal carbon dioxide (mmHg) during VF arrest and basic resuscitation efforts. Figure 1 illustrates gasping during continuous chest compressions. 

Figure 2 illustrates gasping during basic cardiopulmonary resuscitation (CPR) with 2 breaths, before each 30 chest compressions. 

One investigator checked the recorded flow- and EtCO_2_-tracings of all animals. Unclear signals were reviewed and discussed with a second investigator. Gasping was analyzed from the moment of VF induction to first defibrillation.

Data on gasping was available in 61 of the 64 swine. Of those with no data, the capnograph malfunctioned for one animal, and for two animals the data files were missing. 

### 2.1. Statistical Analysis

Data were entered into Microsoft Excel for Windows (Microsoft Corp, Redmond, WA) and were analyzed using SPSS 16.0 for Windows (SPSS, Inc. Chicago, IL). Continuous variables were presented as mean ± SD and median with 25/75 percentiles as appropriate and were analyzed by independent *t*-test, one-way independent ANOVA, Mann-Whitney *U*-test or Kruskal-Wallis test, as indicated. Independent ANOVA was used to assess the difference in gasping frequency among pretreatment groups. Gasping data were not normally distributed among posttreatment groups or among the outcome groups. The results of Levene's test for the treatment groups showed *F* (3, 57) = 7.93, *P* < .001, and for the outcome-VF time groups showed *F*(3, 370) = 9.45, *P* < .001, indicating non-homogeneity among groups. Therefore, the Kruskal-Wallis test was used to detect the effect of treatment upon gasping (Table 2). A Bonferroni correction was applied, and all effects are reported at a significance level of 0.008.

## 3. Results

**Figure 3:**
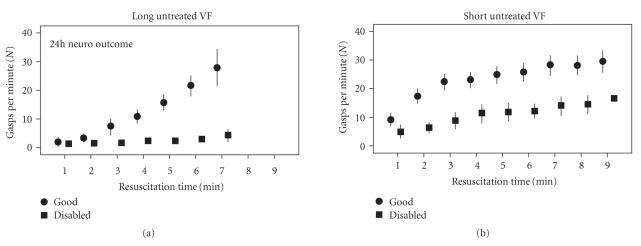
This figure presents the number of gasps per minute plotted against the duration of basic resuscitation efforts. (a) Long Untreated VF plots the number of gasps per minute (mean ± standard error) for each minute of basic resuscitation efforts irrespective of the applied resuscitation type in those with 5 to 6 minutes of untreated VF prior to the onset of basic resuscitation techniques. (b) Short Untreated VF plots the number of gasps per minute (mean ± standard error) for each minute of basic resuscitation efforts irrespective of the applied resuscitation type in those with 3 to 4 minutes of untreated VF prior to the onset of basic resuscitation techniques. Circles indicate those with good 24-hour neurological outcome, and the squares indicate those disabled (death or poor neurological outcomes). Standard error is indicated by the vertical lines.

With basic resuscitation efforts, gasping was more likely to continue or if absent to resume in the animals with short rather than long untreated VF durations (Figure 3). 

During the first 3 minutes of basic resuscitation efforts, the frequency of gasping was greater in the short VF group (median = 11) compared with the long untreated VF group (median = 0), *U* = 82, *P* < .001, *r* = 0.7. It took longer for gasping to resume or appear in the long untreated VF group (Figure 3). Higher gasping rates were significantly and positively associated with good neurological outcome and survival (Figure 3, Tables 1 and 2). 

**Figure 4:**
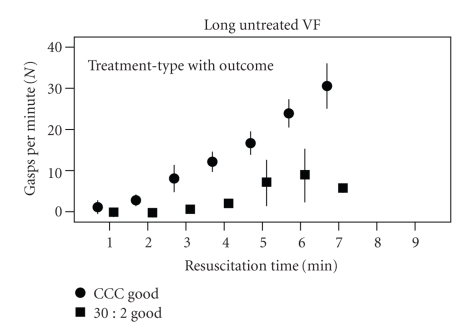
This figure presents the number of gasps per minute (mean ± standard error) plotted against the duration of basic resuscitation efforts in animals with good with good 24 hour neurological outcome neurological outcome. Only those animals with 5 or 6 minutes of untreated VF (Long Untreated VF) prior to the onset of basic resuscitation are shown. Circles indicate those treated with continuous chest compression (CCC) basic resuscitation; squares indicate those treated with 30: 2 basic resuscitation prior to advanced cardiac life support. Standard error is indicated by the vertical lines.

Gasping recurred sooner and was more frequent in animals treated with continuous chest compressions (CCCs) compared with 30 : 2 compressions to ventilations (30 : 2). The slope of increase in gasping frequency was significant (Jonckheere's test: *J* = 1,150, *z* = 5.86, *r* = 0.8). In the animals with good neurological outcome, gasping occurred sooner and was more frequent in the CCC group compared with the 30 : 2 group (Figure 4). 

**Figure 5:**
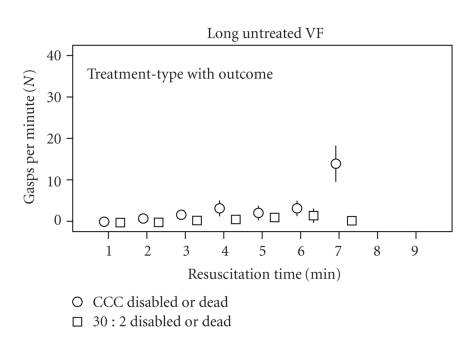
This figure presents the number of gasps per minute (mean ± standard error) plotted against the duration of basic resuscitation efforts in animals disabled or dead at 24 hours. Only those animals with 5 or 6 minutes of untreated VF (Long Untreated VF) prior to the onset of basic resuscitation are shown. Circles indicate those treated with continuous chest compression (CCC) basic resuscitation; squares indicate those treated with 30 : 2 basic resuscitation prior to advanced cardiac life support. Standard error is indicated by the vertical lines.

Animals with poor neurological outcome (disabled or dead) did not have rapid gasping rates (Figure 5).

As shown in Table 1, the total number of gasps during the respective treatment durations was significantly more frequent during the short rather than long untreated VF duration and was more frequent with CCC than with 30 : 2 compressions to ventilations. As shown in Table 1, during each time period, the neurological outcome was better in those with more frequent gasping. Animals that did not resume gasping after the initiation of resuscitation efforts had a worse outcome. 

The mean integrated coronary perfusion pressure during basic life support was 20 ± 10 mmHg with CCC and 14 ± 10 mm Hg with 30 : 2 CPR (*P* < .028). The mean integrated coronary perfusion pressure in the 24-hour survivors with normal neurological function was 23 ± 8 mmHg but only 10 ± 7 mmHg in animals with severe neurological deficits (*P* < .001). There was no difference in the mean integrated coronary perfusion pressure between the survivors with severe neurological deficits and nonsurvivors.

## 4. Discussion

This study confirms the importance of gasping during resuscitation efforts in subjects with primary cardiac arrest due to ventricular fibrillation and found that not only the duration between the arrest and the initiation of basic resuscitation techniques influenced the frequency of gasping but also the type of basic resuscitations technique. This confirms the previous investigations in humans showing a positive association between the presence of gasping and outcome [14]. In patients with OHCA, 39% with witnessed cardiac arrest who were noted to be gasping while receiving CPR survived whereas only 9% of those who did not gasp while receiving CPR survived [14]. The present investigation on cardiac arrest in swine extends these findings, indicating that not only the presence but also the frequency of gasping during resuscitation efforts influences survival, and that the type of basic resuscitation influences the frequency of gasping. 

This study also found that animals that were gasping at the onset of resuscitation efforts were not only likely to continue gasping but were also likely to increase the frequency of gasping (Figure 3; Short Untreated VF). Animals that were no longer gasping, but which resumed gasping following the onset of basic CPR (Figure 3; Long Untreated VF) were more likely to have good neurological outcome. Animals that had stopped gasping and did not resume gasping during resuscitation efforts had a clearly reduced likelihood of good neurological outcome (Figure 5). 

The present study also found that animals with longer untreated VF durations (5 or 6 minutes) required a longer period of basic resuscitation efforts before resumption gasping (Figure 3). The analysis also demonstrated a positive association between the type of basic resuscitation protocol (CCC versus 30 : 2), gasping (CCC versus 30 : 2), gasping, and survival (Figure 4, Table 1). Animals receiving CCC resuscitation increased their rate of gasping faster, achieving a higher frequency per minute compared with those receiving the 30 : 2 compressions to ventilations (Figure 4). Furthermore, these animals were more likely to survive 24 hours with good neurological outcome (Figure 4). 

Noc and associates reported in a rodent model with an untreated VF time of 4 minutes that the initiation of chest compressions without ventilation often resulted in gasping at an increasing rate, and that frequent gasping was associated with improved survival [3, 4]. Similar findings were reported by others [13]. Frenneaux and Steen state that in swine, “if effective cardiac compressions are given to gasping pigs they continue to gasp as long as the chest compressions create a minimum blood flow to the brain stem” [21]. 

It has been shown in experimental models that a major determinant of survival after cardiac arrest is coronary perfusion pressure (i.e., the difference between the aortic and right atrial pressure during the release phase of chest compression) [15]. Uninterrupted chest compressions produced higher integrated coronary perfusion pressure relative to the 30 : 2 CPR group, because of the required pauses in compressions in the latter group to provide breaths. Furthermore, CCC generated more consistent arterial systolic pressures, which provided cerebral perfusion and presumably contributed to the better neurologically normal survival in this group.

The present study found that during VF arrest and resuscitation efforts, that gasping recurred sooner, was more frequent, and was associated with a higher 24-hour neurologically normal survival in animals receiving basic resuscitation efforts with CCC compared with 30 : 2 CPR. 

There are limitations to this study. A major limitation is that in this study of gasping during basic life support, the swine were intubated (an intervention normally reserved for advanced cardiac life support). This is a significant limitation of almost all swine studies of basic life support, as the upper airway of swine is significantly different from that of man. Another limitation of this study is the unusually high systolic pressures generated by chest compressions. A third and fourth limitation is that the chests of these swine were more compliant than would be extant in elderly humans, and the swine did not have cardiovascular disease. Gasping is more pronounced in immature animals [5, 9, 22]. Although the swine used in this study were not immature, they were young, and this may have increased the relative frequency of gasping. Animals that are paralyzed as part of their anesthetic preparation do not gasp. The phenomenon of gasping is also related to the type and duration of anesthesia the animals are subjected to before the induction VF and resuscitation attempts. The swine in this study were anesthetized with isoflurane, which is less likely to suppress gasping relative to some other commonly used anesthetics, but might reduce the respiratory stimulation due to increasing pCO_2_ [23]. In our experience, high doses of anesthetics including isoflurane will also suppress gasping. 

 The findings of the present study suggest that increasing attention needs to be directed at the presence or absence of gasping during cardiac arrest and resuscitation efforts in man. If frequent gasping during chest compressions is misinterpreted as resumption of breathing, the compressions might be prematurely stopped or interrupted. Increasing gasping rates need to be recognized as indicator of effect bystander resuscitation efforts.

## 5. Conclusions

This study, in a swine model of out-of-hospital cardiac arrest due to ventricular fibrillation, confirmed the association of gasping during basic resuscitation efforts and survival. In addition, it was observed that gasping was more likely to continue or if absent to resume in the swine with 3 to 4 minutes of untreated VF before the initiation of basic resuscitation. With longer durations, 5 to 6 minutes of untreated VF before basic resuscitation efforts, gasping was more frequent and survival better in those receiving continuous chest compression basic resuscitation. In the absence of frequent gasping, intact survival was rare in this subgroup.

## 6. Conflict of Interest and Support

Mathias Zuercher has significant support from the “Anaesthesieverein” of the Department of Anesthesia and Intensive Care, University Hospital Basel, Switzerland. G. A. Ewy has an unrestricted grant from private donations to the University of Arizona Foundation for support of Sarver Heart Center's Resuscitation Research and is a co-investigator on an unrestricted grant from the Laerdal Foundation of Stavanger, Norway. Both grants are significant. R. A. Berg has grants from the National Institutes of Health, National Heart, Lung, and Blood Institute, Bethesda, MD, and from Medtronics Inc., Minneapolis, MN; all grants are significant. K. B. Kern is the principal investigator of a significant unrestricted grant from the Laerdal Foundation of Stavanger, Norway, and is on the Scientific Advisory committee of Zoll Inc., Chelmsford, MA and Physio-Control Inc., Redmond, WA; both are not significant.

## Figures and Tables

**Table 1:** (a) Frequency of gasping in relation to the resuscitation protocol applied. (b) Frequency of gasping in relation to the 24-hour neurological outcome.

				Frequency	
Untreated VF time	Groups	*N*	Mean (SD)	Median (P25–75)	Kruskal-Wallis test
(a) **By Treatment**					H (3) = 30.7,
5 and 6 minutes	30 : 2	16	1.1 (2.1)	0.07 (0–1.3)	*P* < .001
	CCC	15	8.4 (6.7)	6.4 (0.8–15.6)	
3 and 4 minutes	30 : 2	14	15.4 (9.8)	16.1 (6.8–16.1)	
	CCC	16	21.1 (10.9)	21.2 (12.8–31.2)	
b) **By Outcome**					H (3) = 38.8,
5 and 6 minutes	Disabled/dead	19	1.1 (2.2)	0.14 (0–0.8)	*P* < .001
	Good	12	10.2 (6.1)	9.6 (3.1–16.0)	
3 and 4 minutes	Disabled/dead	9	10.1 (7.4)	9.8 (5.3–9.6)	
	Good	21	21.2 (9.9)	22.0 (14.9–22.0)	

Posthoc analysis

*N *: number of animals per group; SD: standard deviation; P25–75: 25th–75th percentile.

Frequency = number of spontaneous ventilations or gasps per minute. Mann-Whitney tests with exact significance levels and Bonferroni correction, *P* = .008).

**Table 2:** (a) Frequency of gasping in relation to the resuscitation protocol applied. (b) Frequency of gasping in relation to the 24-hour neurological outcome.

Reference group	Comparison VF group	Mann-Whitney *U*-test
(a)** By treatment**		
5 or 6 min VF—30 : 2	5-or 6-minute VF—CCC	*U* = 34, *P* < .001, *r* = −0.6
	3-or 4-minute VF—30 : 2	*U* = 17, *P* < .001, r = −0.7
	3-or 4-minute VF—CCC	*U* = 13, *P* < .001, r = −0.7
5 or 6 min VF—CCC	3-or 4-minute VF—30 : 2	*U* = 59, *P* = .04, *r* = −0.4
	3-or 4-minute VF—CCC	*U* = 42, *P* = .001, *r* = −0.6
3 or 4 min VF—30 : 2	3-or 4-minute VF—CCC	*U* = 80, *P* = .18, *r* = −0.3
(b) **By outcome**		
5 or 6 min VF—Disabled/dead	3-or 4-minute VF—Disabled/dead	*U* = 30, *P* = .005, *r* = −0.5
	5-or 6-minute VF—Good	*U* = 11, *P* < .001, *r* = −0.8
	3-or 4-minute VF—Good	*U* = 6, *P* < .001, *r* = −0.8
3 or 4 min VF—Disabled/Dead	5-or 6-minute VF—Good	*U* = 54, *P* = 1.0, *r* = 0
	3-or 4-minute VF—Good	*U* = 31, *P* = .004, *r* = −0.5
5 or 6 min VF—Good	3-or 4-minute VF—Good	U = 40, *P* = .001, r = − 0.6

Posthoc analysis

*N*: number of animals per group; SD: standard deviation; p25–75: 25th–75th percentile.

Frequency = number of spontaneous ventilations or gasps per minute. Mann-Whitney tests with exact significance levels and Bonferroni correction, *P* = .008.
